# Effectiveness of a novel and scalable clinical decision support intervention to improve venous thromboembolism prophylaxis: a quasi-experimental study

**DOI:** 10.1186/1472-6947-12-92

**Published:** 2012-08-31

**Authors:** Craig A Umscheid, Asaf Hanish, Jesse Chittams, Mark G Weiner, Todd EH Hecht

**Affiliations:** 1 Center for Evidence-based Practice, University of Pennsylvania, Suite 50 Mezzanine, 3535 Market Street, Philadelphia, PA, USA; 2 Department of Medicine, University of Pennsylvania, 3535 Market Street, Mezzanine, Suite 50, Philadelphia, PA, USA; 3 Center for Clinical Epidemiology and Biostatistics, University of Pennsylvania, 3535 Market Street, Mezzanine Suite 50, Philadelphia, PA, USA; 4 Leonard Davis Institute of Health Economics, University of Pennsylvania, 3535 Market Street, Mezzanine, Suite 50, Philadelphia, PA, USA

**Keywords:** Electronic medical record, Electronic health record, Health information technology, Clinical decision support, Venous thrombosis, Deep venous thrombosis, Quasi-experimental study

## Abstract

**Background:**

Venous thromboembolism (VTE) causes morbidity and mortality in hospitalized patients, and regulators and payors are encouraging the use of systems to prevent them. Here, we examine the effect of a computerized clinical decision support (CDS) intervention implemented across a multi-hospital academic health system on VTE prophylaxis and events.

**Methods:**

The study included 223,062 inpatients admitted between April 2007 and May 2010, and used administrative and clinical data. The intervention was integrated into a commercial electronic health record (EHR) in an admission orderset used for all admissions. Three time periods were examined: baseline (period 1), and the time after implementation of the first CDS intervention (period 2) and a second iteration (period 3). Providers were prompted to accept or decline prophylaxis based on patient risk. Time series analyses examined the impact of the intervention on VTE prophylaxis during time periods two and three compared to baseline, and a simple pre-post design examined impact on VTE events and bleeds secondary to anticoagulation. VTE prophylaxis and events were also examined in a prespecified surgical subset of our population meeting the public reporting criteria defined by the Agency for Healthcare Research and Quality (AHRQ) Patient Safety Indicator (PSI).

**Results:**

Unadjusted analyses suggested that “recommended”, “any”, and “pharmacologic” prophylaxis increased from baseline to the last study period (27.1% to 51.9%, 56.7% to 78.1%, and 42.0% to 54.4% respectively; p < 0.01 for all comparisons). Results were significant across all hospitals and the health system overall. Interrupted time series analyses suggested that our intervention increased the use of “recommended” and “any” prophylaxis by 7.9% and 9.6% respectively from baseline to time period 2 (p < 0.01 for both comparisons); and 6.6% and 9.6% respectively from baseline to the combined time periods 2 and 3 (p < 0.01 for both comparisons). There were no significant changes in “pharmacologic” prophylaxis in the adjusted model. The overall percent of patients with VTE increased from baseline to the last study period (2.0% to 2.2%; p = 0.03), but an analysis excluding patients with VTE “present on admission” (POA) demonstrated no difference in events (1.3% to 1.3%; p = 0.80). Overall bleeds did not significantly change. An analysis examining VTE prophylaxis and events in a surgical subset of patients defined by the AHRQ PSI demonstrated increased “recommended”, “any”, and “pharmacologic” prophylaxis from baseline to the last study period (32.3% to 60.0%, 62.8% to 85.7%, and 47.9% to 63.3% respectively; p < 0.01 for all comparisons) as well as reduced VTE events (2.2% to 1.7%; p < 0.01).

**Conclusions:**

The CDS intervention was associated with an increase in “recommended” and “any” VTE prophylaxis across the multi-hospital academic health system. The intervention was also associated with increased VTE rates in the overall study population, but a subanalysis using only admissions with appropriate POA documentation suggested no change in VTE rates, and a prespecified analysis of a surgical subset of our sample as defined by the AHRQ PSI for public reporting purposes suggested reduced VTE. This intervention was created in a commonly used commercial EHR and is scalable across institutions with similar systems.

## Background

Venous thromboembolism (VTE) is an important cause of morbidity and mortality in hospitalized patients. For example, among greater than 7 million patients discharged from 944 US acute care hospitals, postoperative deep venous thrombosis (DVT) was the second most common complication, second most common cause of prolonged hospitalization, and the third most common cause of excess mortality and charges [1].

Without VTE prophylaxis, the incidence of *asymptomatic* hospital-acquired DVT is as high as 20% among medical patients, 40% among general surgical patients, and 60% among orthopedic surgery patients [1]. Rates of *symptomatic* DVT and pulmonary embolism (PE) without pharmacologic prophylaxis are lower at approximately 1% and 1.2% respectively when measured during the hospital stay for nonsurgical patients [2], and 2.8% and 1.5% respectively at 35 days post-op for patients undergoing major orthopedic surgery [3]. Pharmacologic prophylaxis decreases *symptomatic* DVT and PE by approximately 25% and 30% respectively in nonsurgical patients [2], and approximately 55% and 60% respectively in major orthopedic surgery patients [3].

Given the impact of VTE on hospital inpatients, and the effectiveness of prophylaxis at reducing VTE events, hospital regulators and payors are beginning to incentivize hospitals to implement systems changes to prevent VTE. For example, in Fiscal Year 2009, Medicare stopped providing additional reimbursements for DVT and PE following hip or knee replacement [4,5]. Many methods to improve VTE prophylaxis in the hospital setting have been studied, including the use of computerized clinical decision support (CDS) interventions [6]. However, many of the CDS interventions studied were limited in that they either used noncommercial homegrown electronic health records (EHR) [7-13], pop-up alerts to remind providers when patients with VTE risk factors were not on prophylaxis [7-11,13-16], or targeted select populations [8,11,12] or individual hospitals [17]. These limitations could impact the generalizability of the studies’ findings across multi-hospital systems, particularly given the ubiquitous nature of pop-up alerts and the associated alert fatigue [18], challenges associated with translating the functionality of a homegrown EHR into a commercial one, and obstacles associated with implementing change across multiple institutions. In this study, we examine the effect on VTE prophylaxis and event rates of integrating a CDS intervention that does not involve pop-up alerts into a commonly used commercial EHR serving all inpatients in a multi-hospital academic health system.

## Methods

### Developing and implementing the CDS

The CDS intervention was developed, implemented and tested in a quaternary care academic health system comprised of three acute care teaching hospitals: the 814 bed flagship hospital (Hospital A), a 331 bed hospital best known for its services in cardiology, ophthalmology, orthopedics, family medicine, geriatrics and psychiatry (Hospital B), and a 569 bed hospital best known for its services in orthopedics, obstetrics and neurosurgery (Hospital C). Overall, these three hospitals include 1,714 beds that are staffed by more than 4,000 attending physicians, residents and fellows. The hospitals account for approximately 80,000 admissions annually.

We created a CDS intervention that was linked to an electronic admission order set in a commercial EHR (Sunrise Clinical Manager 5.0, Allscripts, Chicago, IL) used by these three hospitals. The intervention was designed to improve VTE risk assessment and prophylaxis across these hospitals. Recommendations for prophylaxis were based on locally adapted national guidelines [1,19]. Key participants in the development and implementation of the intervention were physicians, nurses, quality specialists, pharmacists, informatics analysts, and anticoagulation experts.

Before launching the intervention across the health system, pharmacists increased their stock of VTE prophylaxis, and nurses increased the supply of mechanical VTE prophylaxis on the wards. Nurses also implemented a nursing education program about the intervention and the availability of low molecular weight heparin (LMWH) as a VTE prophylaxis option. In addition, an email communication about the intervention was distributed to all faculty and staff. Lastly, a point-of-use educational video was developed and implemented to educate ordering providers about the intervention.

**Figure 1:**
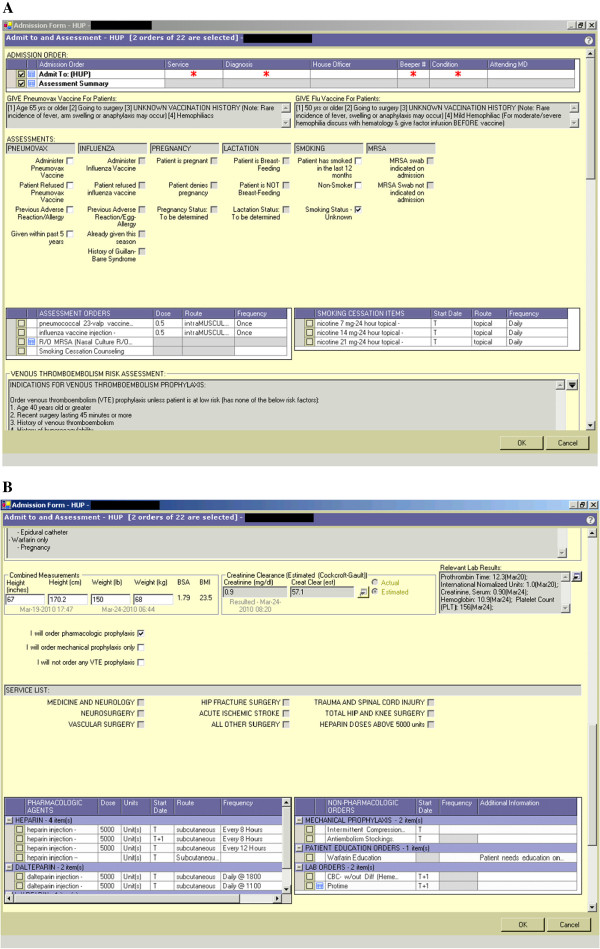
**Screenshots of clinical decision support intervention. A**. Admission order set containing VTE prophylaxis risk assessment; **B**. VTE prophylaxis order grid.

The first iteration was launched at Hospital A and Hospital B on 4/7/08, and at Hospital C on 4/8/08. This version required the admitting provider to accept or decline VTE prophylaxis based on patient risk. Providers were expected to order prophylaxis unless their patients were low risk as defined by the absence of eleven listed risk factors (1. age ≥40, 2. recent surgery lasting ≥ 45 minutes, 3. history of venous thromboembolism, 4. history of hypercoagulability, 5. history of cancer, 6. obesity (BMI ≥ 30), 7. ongoing estrogen or anti-androgen use, 8. history of varicose veins, 9. reduced mobility, 10. weakness or paralysis of ≥ one limb, 11. expected length of stay ≥ 3 days). Because it was rare for a patient to have none of the eleven listed risk factors, we did not ask providers to check off risk factors as part of the ordering process, nor did the CDS auto-populate risk factors based on the medical record. If prophylaxis was declined, a reason had to be specified using a drop down menu, which included a set of pre-defined reasons (e.g. patient has none of the listed risk factors, patient is on therapeutic anticoagulation) as well as an option to insert free text. If prophylaxis was accepted, a list of contraindications to pharmacologic prophylaxis was displayed and included: active or recent bleeding, known bleeding disorder or coagulopathy (INR 2 or greater), patient on therapeutic anticoagulation with heparin or warfarin, platelet count less than 50,000; for heparins, history of heparin-induced thrombocytopenia or allergy to heparin; for low molecular weight heparins, creatinine clearance less than 30 mL/min or epidural catheter; and for warfarin, pregnancy. The user could then enter the admission order and proceed to a separate VTE prophylaxis order set that would provide guidance to the most appropriate prophylaxis based on the clinical service. A second iteration of the CDS, launched across the health system on 9/15/09, combined the VTE risk assessment and prophylaxis order grid into one intervention embedded in the electronic admission orderset. Providers were then confronted with three options instead of two: 1) pharmacologic prophylaxis; 2) mechanical prophylaxis only; or 3) no prophylaxis. The last two options required a reason to be specified. When a user selected the “pharmacologic prophylaxis” option, the order grid appeared and would automatically populate based on the service selected by the user (Figure 1A and B). The second iteration also included an updated list of risk factors, pharmacologic contraindications, and pre-defined reasons to decline prophylaxis. Both versions of the intervention included logic to prevent two anticoagulants from being ordered at once, and a glomerular filtration rate (GFR) calculator to prevent the use of LMWH in those with a creatinine clearance less than 30 cc/min.

### Study subjects

The retrospective study was approved by the University of Pennsylvania Institutional Review Board with a HIPAA waiver. Study subjects included all adults admitted to an acute care inpatient service at our academic health system. Those in our original dataset without a listed discharging service were excluded. The first study period included admissions in the 12 months prior to the first CDS intervention; the second period included admissions between the first and second versions of the CDS intervention; the third period included admissions in the eight months following the implementation of the revised CDS intervention (9/15/09-5/31/10 for Hospital A, Hospital B, and Hospital C).

### Data sources and measures

Clinical data was retrieved from the inpatient EHR, and administrative data was retrieved from Horizon Performance Manager (HPM), which included professionally coded data from the medical record.

Measures included descriptive characteristics of patients (age, gender, race, insurance status, length of stay, weight, creatinine clearance as estimated by the Cockcroft-Gault equation, discharging unit and hospital, primary and secondary discharge diagnostic codes, and severity of illness as measured by diagnostic related group [DRG] weight), CDS utilization measures (including provider response to the CDS intervention [i.e., a “yes” versus “no” response to the question of whether or not the provider would order VTE prophylaxis] as well as selected reasons for declining prophylaxis), and VTE process and outcome measures. Inpatient services were categorized into “medicine” services (neurology, allergy, cardiology, endocrinology/diabetes, family medicine, gastroenterology, general internal medicine, geriatrics, hematology/oncology, hospitalists, infectious diseases, neurology, pulmonary, renal/metabolic and rheumatology), “orthopedic/trauma” services, and the “other surgery” services (anesthesia, cardiac surgery, colorectal surgery, obstetrics, gynecological oncology, gastrointestinal surgery, maternal fetal medicine, neurosurgery, otolaryngology, plastic surgery, general surgery, surgical oncology, thoracic surgery, transplant surgery, urology, oral/maxillofacial, and vascular surgery). VTE process measures included percent of patients on *recommended prophylaxis* (per our guideline), *any prophylaxis* (i.e., pharmacologic or mechanical), and *pharmacologic prophylaxis* (i.e., unfractionated heparin, LMWH, or warfarin). *Recommended prophylaxis* was defined as enoxaparin 30 mg twice daily, dalteparin 5,000U once daily or warfarin for the orthopedics/trauma services; and unfractionated heparin (UFH) 5,000U thrice daily, enoxaparin 40 mg once daily, or dalteparin 5000U once daily for the medicine services and other surgery services. *Any prophylaxis* was defined as any pharmacologic prophylaxis or mechanical prophylaxis (i.e. intermittent pneumatic compression devices). *Any pharmacologic prophylaxis* was defined as any standard prophylactic UFH or LMWH dose, or any warfarin dose for the orthopedics/trauma services. Unless otherwise stated, patients were judged to receive pharmacologic prophylaxis if they were administered at least one ordered dose. Mechanical prophylaxis was defined by order only. Clinical outcome measures included VTE and bleeding events during hospitalization. VTE events were defined as any hospital discharge with a secondary discharge diagnosis of PE or DVT as defined by the *International Classification of Diseases Version 9* (ICD9) codes listed in the Agency for Healthcare Research and Quality (AHRQ) Patient Safety Indicators (PSI) Technical Specifications Guide under PSI 12 [20]. Discharges that included the PE or DVT codes as primary discharge diagnoses were excluded. The AHRQ definition of VTE was applied to the overall study population, as well as a prespecified surgical subset of our study population who met the AHRQ PSI criteria for public reporting of VTE events [20]. A subanalysis was performed excluding patients with VTE codes listed as “present on admission” (POA). POA data was only available for hospitalizations after 10/1/07 at Hospital A, 11/7/07 at Hospital C, and 4/1/08 at Hospital B. Bleeding was defined as any one of a set of secondary discharge diagnosis codes related to bleeding [21] combined with any one of a set of E codes representing medical or drug errors (E858.1, E858.2, E858.8, E858.9, E876.8, E876.9, E934.2, E934.3, E934.8, E947.8, E947.9, E980.4, E980.5).

### Data analysis

Unadjusted analyses using chi-square tests for dichotomous variables and t tests for continuous variables compared demographics and the proportion of VTE process and outcome measures across the three time periods. Unadjusted analyses using chi-square tests were also used to compare over our two follow-up periods the proportion of providers who ordered prophylaxis when they indicated in the CDS that they would do so, as well as the proportion of providers who did not order prophylaxis when they indicated in the CDS that they would not do so. In addition, proportions were calculated for the reasons noted or selected for declining prophylaxis.

Adjusted analyses using interrupted time series models estimated the impact of the original and revised CDS interventions on administration of VTE prophylaxis. These analyses were employed using the SAS/ETS procedure PROC AUTOREG with the CHOW option (SAS, 1999). This type of analysis allowed us to measure the impact of the CDS interventions while adjusting for the serial correlation among hospital level data collected sequentially in time as well as potential unmeasured confounders, such as other quality interventions that could have impacted VTE prophylaxis [22].

## Results

The study included 223,062 inpatients across our three hospitals in the study time period (between April 2007 and May 2010), and excluded 1546 encounters (0.70% of the total encounters in our dataset) who did not have a discharge service listed. The baseline characteristics of the patient populations across our three time periods were clinically similar (Table 1).

**Table 1:** Descriptive statistics of patient population across study time periods

**Variable**		**Period 1**	**Period 2**	**Period 3**	**P value**
Age (years)	Mean (SD)	53.6 (19.1)	53.6 (19.1)	54.0 (18.9)	<0.01
Sex	Female	40,333 (57.4%)	58,506 (57.1%)	28,799 (57.2%)	0.56
Race	White	38,317 (54.5%)	56,095 (54.8%)	27,039 (53.7%)	<0.01
	Other	31,547 (44.9%)	45,984 (44.9%)	23,109 (45.9%)	
	Unknown	419 (0.6%)	329 (0.3%)	223 (0.4%)	
Health Insurance	Self Pay	841 (1.2%)	1,258 (1.2%)	501 (1.0%)	<0.01
	Insured	65,246 (92.8%)	99,895 (97.5%)	49,307 (97.9%)	
	Unknown	4,196 (6.0%)	1,255 (1.2%)	563 (1.1%)	
Weight (kg)	Mean (SD)	82.3 (24.0)	82.8 (24.1)	82.8 (24.1)	<0.01
CrCl <30	Yes	5,159 (9.7%)	7,547 (9.9%)	3,828 (8.8%)	<0.01
	No	47,855 (90.3%)	68,743 (90.1%)	39,513 (91.2%)	
Length of Stay (days)	Mean (SD)	5.2 (7.7)	5.1 (7.6)	5.2 (7.0)	<0.01
DRG Weight	Mean (SD)	1.7 (1.9)	1.7 (1.9)	1.7 (2.0)	<0.01
Discharging Unit	Medicine	2,488 (46.2%)	47,973 (46.8%)	24,373 (48.4%)	<0.01
	Ortho/Trauma	7,657 (10.9%)	11,506 (11.2%)	5,825 (11.6%)	
	Other Surgery	30,138 (42.9%)	42,929 (41.9%)	20,173 (40.0%)	
Top 5 Primary Discharge Diagnoses	First	Device complications 2,856 (4.4%)	Device complications 4,423 (4.5%)	Device complications 2,355 (4.8%)	NA
	Second	Osteoarthritis 2,327 (3.6%)	Osteoarthritis 3,505 (3.5%)	Osteoarthritis 1,805 (3.7%)	
	Third	CAD 2,104 (3.3%)	CAD 3,342 (3.4%)	Dysrhythmia 1,676 (3.5%)	
	Fourth	CHF 1,971 (3.0%)	Dysrhythmia 3,321 (3.4%)	CHF 1,460 (3.0%)	
	Fifth	Dysrhythmia 1,940 (3.0%)	Birth complications 2,935 (3.0%)	CAD 1,419 (2.9%)	

Abbreviations: CAD, coronary artery disease; CHF, congestive heart failure; CrCl, creatinine clearance; DRG, diagnosis-related group; SD, standard deviation.

**Figure 2:**
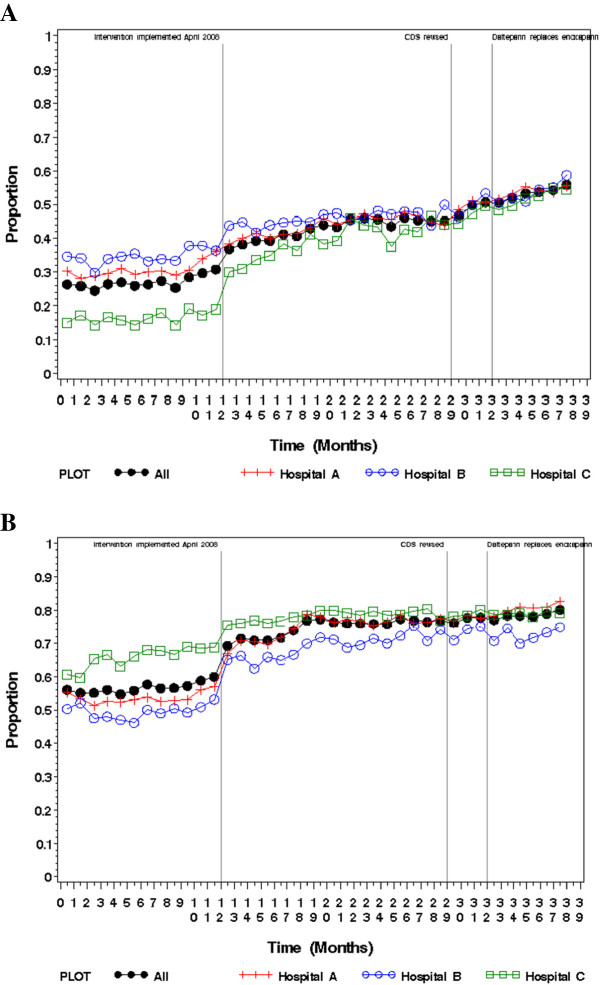
**Proportion of patients receiving VTE prophylaxis by month. A**. Recommended VTE prophylaxis; **B**. Any VTE prophylaxis.

In our unadjusted analyses, “recommended” prophylaxis significantly increased across the three study periods across all hospitals and services (27.1% vs. 43.0% vs. 51.9%; p < 0.01). (Table 2 and Figure 2A) “Other surgical services” had the greatest increase overall from 19.8% to 38.5% to 48.2% (p < 0.01). The orthopedics/trauma services had the lowest rate of increase overall, from 44.4% to 46.4% to 48.8% (p < 0.01), but had the highest rate of “recommended prophylaxis” before the CDS interventions. The administration of “any” prophylaxis increased as well across all hospitals and services (56.7% vs. 74.7% vs. 78.1%; p < 0.01) (Table 3 and Figure 2B). The orthopedics/trauma services had the highest baseline rate of “any” prophylaxis at 65.9%, which increased to 91.5% and 97.0% (p < 0.01) after the first and second iterations of the CDS interventions respectively. “Pharmacologic” prophylaxis also increased across each of our hospitals and the health system overall (42.0% vs. 47.6% vs. 54.4%; p < 0.01) (Table 4). “Other surgical services” had the greatest increase overall from 33.3% to 42.1% to 49.2% (p < 0.01).

**Table 2:** Change in administration of recommended prophylaxis by hospital and service across the three study time periods

**Hospital**	**Discharge service**	**Period 1**	**Period 2**	**Period 3**	**P-value**
Hospital A	Medicine	5,614 (36.7%)	10,623 (46.2%)	6,491 (54.6%)	<0.01
	Ortho/Trauma	659 (32.1%)	1,215 (35.2%)	746 (40.1%)	<0.01
	Other Surgery	4,372 (25.1%)	10,856 (42.9%)	6,096 (52.5%)	<0.01
	All	10,645 (30.6%)	22,694 (43.9%)	13,333 (52.5%)	<0.01
Hospital B	Medicine	3,460 (35.6%)	6,648 (45.7%)	3,661 (53.2%)	<0.01
	Ortho/Trauma	690 (34.5%)	1,323 (42.9%)	709 (44.1%)	<0.01
	Other Surgery	584 (30.8%)	1,393 (50.4%)	771 (58.6%)	<0.01
	All	4,734 (34.8%)	9,364 (45.9%)	5,141 (52.4%)	<0.01
Hospital C	Medicine	581 (7.8%)	4,897 (47.0%)	3,411 (61.0%)	<0.01
	Ortho/Trauma	2,052 (57.0%)	2,797 (56.3%)	1,390 (58.9%)	0.10
	Other Surgery	1,005 (9.3%)	4,265 (28.7%)	2,847 (39.3%)	<0.01
	All	3,638 (16.6%)	11,959 (39.5%)	7,648 (50.3%)	<0.01
Overall	Medicine	9,655 (29.7%)	22,168 (46.2%)	13,563 (55.6%)	<0.01
	Ortho/Trauma	3,401 (44.4%)	5,335 (46.4%)	2,845 (48.8%)	<0.01
	Other Surgery	5,961 (19.8%)	16,514 (38.5%)	9,714 (48.2%)	<0.01
	All	19,017 (27.1%)	44,017 (43.0%)	26,122 (51.9%)	<0.01

**Table 3:** Change in administration of any prophylaxis by hospital and service across the three study time periods

**Hospital**	**Discharge service**	**Period 1**	**Period 2**	**Period 3**	**P-value**
Hospital A	Medicine	8,021 (52.4%)	15,405 (67.0%)	8,655 (72.8%)	<0.01
	Ortho/Trauma	1,275 (62.0%)	3,035 (87.9%)	1,808 (97.2%)	<0.01
	Other Surgery	9,344 (53.7%)	20,273 (80.2%)	9,726 (83.7%)	<0.01
	All	18,640 (53.6%)	38,713 (74.8%)	20,189 (79.6%)	<0.01
Hospital B	Medicine	4,937 (50.8%)	9,306 (64.0%)	4,510 (65.5%)	<0.01
	Ortho/Trauma	826 (41.3%)	2,810 (91.0%)	1,548 (96.4%)	<0.01
	Other Surgery	981 (51.7%)	1,989 (71.9%)	1,088 (82.7%)	<0.01
	All	6,744 (49.6%)	14,105 (69.1%)	7,146 (72.9%)	<0.01
Hospital C	Medicine	5,553 (74.3%)	8,123 (77.9%)	4,445 (79.4%)	<0.01
	Ortho/Trauma	2,947 (81.8%)	4,682 (94.3%)	2,294 (97.3%)	<0.01
	Other Surgery	5,932 (54.8%)	10,878 (73.1%)	5,257 (72.7%)	<0.01
	All	14,432 (65.9%)	23,683 (78.3%)	11,996 (79.0%)	<0.01
Overall	Medicine	18,511 (57.0%)	32,834 (68.4%)	17,610 (72.3%)	<0.01
	Ortho/Trauma	5,048 (65.9%)	10,527 (91.5%)	5,650 (97.0%)	<0.01
	Other Surgery	16,257 (53.9%)	33,140 (77.2%)	16,071 (79.7%)	<0.01
	All	39,816 (56.7%)	76,501 (74.7%)	39,331 (78.1%)	<0.01

**Table 4:** Change in administration of pharmacologic prophylaxis by hospital and service across the three study time periods

**Hospital**	**Discharge service**	**Period 1**	**Period 2**	**Period 3**	**P-value**
Hospital A	Medicine	6,753 (44.1%)	10,977 (47.7%)	6,662 (56.0%)	<0.01
	Ortho/Trauma	1,121 (54.5%)	2,207 (63.9%)	1,301 (69.9%)	<0.01
	Other Surgery	6,623 (38.0%)	11,638 (46.0%)	6,162 (53.0%)	<0.01
	All	14,497 (41.7%)	24,822 (48.0%)	14,125 (55.7%)	<0.01
Hospital B	Medicine	4,455 (45.9%)	7,136 (49.0%)	3,792 (55.1%)	<0.01
	Ortho/Trauma	710 (35.5%)	1,355 (43.9%)	730 (45.5%)	<0.01
	Other Surgery	911 (48.0%)	1,563 (56.5%)	800 (60.8%)	<0.01
	All	6,076 (44.6%)	10,054 (49.3%)	5,322 (54.3%)	<0.01
Hospital C	Medicine	4,330 (57.9%)	6,067 (58.2%)	3,538 (63.2%)	<0.01
	Ortho/Trauma	2,103 (58.4%)	2,902 (58.4%)	1,463 (62.0%)	<0.01
	Other Surgery	2,498 (23.1%)	4,862 (32.7%)	2,968 (41.0%)	<0.01
	All	8,931 (40.8%)	13,831 (45.7%)	7,969 (52.5%)	<0.01
Overall	Medicine	15,538 (47.8%)	24,180 (50.4%)	13,992 (57.4%)	<0.01
	Ortho/Trauma	3,934 (51.4%)	6,464 (56.2%)	3,494 (60.0%)	<0.01
	Other Surgery	10,032 (33.3%)	18,063 (42.1%)	9,930 (49.2%)	<0.01
	All	29,504 (42.0%)	48,707 (47.6%)	27,416 (54.4%)	<0.01

Table 5A and B contain the adjusted estimates from the interrupted time series regression models of the effects of the two versions of the CDS intervention on mean monthly percentage of patients receiving “recommended” and “any” prophylaxis by individual hospital and hospital system. The estimates suggest the intervention increased the use of “recommended” and “any” prophylaxis at all three hospitals when comparing the baseline time period 1 with time period 2, and when comparing the baseline time period 1 with the combined intervention periods 2 and 3 (p-values < 0.01). The differences between the intervention periods 2 and 3 were not statistically significant. Our adjusted estimates suggest that the CDS intervention did not significantly increase the use of “pharmacologic” prophylaxis overall or at the level of the hospital across any of the time periods examined in the model.

**Table 5:** Estimated increase in VTE prophylaxis use secondary to the clinical decision support

								
**A. From time period 1 to time period 2**								
	Hospital A		Hospital B		Hospital C		Overall	
	Baseline	Increase	Baseline	Increase	Baseline	Increase	Baseline	Increase
Recommended (%)	31.5	3.2 (p=0.09)	34.5	6.7 (p<.01)	16.4	13.9 (p<.01)	27.1	7.9 (p<.01)
Any Prophylaxis (%)	55.0	10.3 (p<.01)	49.5	13.0 (p<.01)	65.5	6.6 (p<.01)	57.2	9.6 (p<.01)
**B. From time period 1 to time periods 2 and 3**								
	Hospital A		Hospital B		Hospital C		Overall	
	Baseline	Increase	Baseline	Increase	Baseline	Increase	Baseline	Increase
Recommended (%)	30.9	3.7 (p=0.03)	34.5	5.4 (p=0.01)	16.4	13.6 (p<.01)	27.2	6.6 (p<.01)
Any Prophylaxis (%)	55.3	10.4 (p<.01)	49.5	14.6 (p<.01)	65.5	7.2 (p<.01)	57.5	9.6 (p<.01)

Abbreviations: VTE, venous thromboembolism.

To better understand the changes in pharmacologic and mechanical prophylaxis occurring as a result of the CDS interventions, we examined the change in administration of specific prophylaxis regimens by service and hospital. In general, there was a marked reduction in “no prophylaxis” for all services, most prominently orthopedics/trauma, which decreased its overall “no prophylaxis” rate from 34.1% to 8.5% to 3.0% across the three study periods (p < 0.01). The greatest increase in prophylaxis on the orthopedics/trauma services was mechanical prophylaxis, which increased overall from 14.5% to 35.3% to 37.0% (p < 0.01) across the three study periods. There were minor increases in recommended pharmacologic prophylaxis on the orthopedics/trauma services as well, including an increase in appropriate LMWH usage from 15.7% to 19.1% to 18.8% (p < 0.01). On the medicine services, there were significant changes in the types of pharmacologic prophylaxis ordered by providers. The use of twice daily UFH was reduced from 17.5% to 3.6% to 1.6% (p < 0.01) across the three time periods in favor of recommended pharmacologic prophylaxis regimens including thrice daily UFH (increased from 29.2% to 38.2% to 51.0%, p < 0.01) and LMWH (increased from 0.4% to 7.8% to 4.5%, p < 0.01). Increases in thrice daily UFH were most pronounced at Hospital C (increasing from 6.6% to 38.9% to 58.0%, p < 0.01). The “other surgical” services also reduced their rates of “no prophylaxis” from 46.1% to 22.8% to 20.3% (p < 0.01) in favor of sharp increases in the use of thrice daily UFH (from 19.5% to 37.9% to 47.5%, p < 0.01). Unlike the medicine services, the “other surgical” services did not have a clinically significant increase in the amount of LMWH administered (0.5% to 0.7% to 0.7%, p < 0.01).

There was a significant increase in VTE in the overall study population (2.0% vs. 2.1% vs. 2.2%; p = 0.02); however, subanalyses excluding VTE that were “present on admission” suggested no difference in VTE events across the three time periods (1.3% vs. 1.3% vs. 1.3%; p = 0.80). The number of patients with documented bleeds related to anticoagulation was low at 0.1% across all three time periods.

An analysis of VTE prophylaxis and events in a surgical subset of patients defined by the AHRQ PSI used for public reporting demonstrated increased “recommended,” “any,” and “pharmacologic” prophylaxis from baseline to the last study period (32.3% vs. 50.7% vs. 60.0%, 62.8% vs. 82.0% vs. 85.7%, and 47.9% vs. 56.3% vs. 63.3%, respectively; p < 0.01 for all comparisons) as well as a significant reduction in DVT and overall VTE events, but a significant increase in PE (Table 6).

**Table 6:** Change in VTE as defined by AHRQ PSI by hospital across the study time periods

	***Period 1***	***Period 2***	***Period 3***	***P Value***
**Hospital A**				
DVT	2.27 (%)	2.27 (%)	1.56 (%)	<0.01
PE	0.68 (%)	0.71 (%)	0.80 (%)	0.34
DVT/PE	2.82 (%)	2.80 (%)	2.18 (%)	<0.01
**Hospital B**				
DVT	1.05 (%)	0.88 (%)	0.44 (%)	<0.01
PE	0.42 (%)	0.41 (%)	0.71 (%)	<0.01
DVT/PE	1.42 (%)	1.20 (%)	1.09 (%)	0.06
**Hospital C**				
DVT	1.64 (%)	1.67 (%)	1.08 (%)	<0.01
PE	0.29 (%)	0.27 (%)	0.62 (%)	<0.01
DVT/PE	1.83 (%)	1.86 (%)	1.48 (%)	0.10
**Overall**				
DVT	1.77 (%)	1.75 (%)	1.15 (%)	<0.01
PE	0.52 (%)	0.53 (%)	0.74 (%)	<0.01
DVT/PE	2.18 (%)	2.15 (%)	1.73 (%)	<0.01

Abbreviations: AHRQ PSI, Agency for Healthcare Research and Quality Patient Safety Indicator; DVT, deep venous thrombosis; PE, pulmonary embolism; VTE, venous thromboembolism.

When we examined the type of prophylaxis administered stratified by how the provider initially responded to the CDS intervention (i.e., a “yes” versus “no” response to the question of whether or not the provider would order VTE prophylaxis), we found that most providers who indicated they would order prophylaxis did order it, and that proportion increased over our two follow-up periods, from 89.0% to 93.8% in periods 2 and 3, respectively (p < 0.01). Those who said they wouldn't order prophylaxis often did not (63.7% ordered no prophylaxis in period 2 vs. 74.1% in period 3, p < 0.01). The most commonly stated reasons for not ordering prophylaxis were that the patient 1) had no risk factors (58%), 2) was on therapeutic anticoagulation (35%), 3) was peri-procedure (4%), or 4) was a bleeding risk (2%).

## Discussion

The increases in “recommended” and “any” VTE prophylaxis attributable to the CDS intervention evaluated in our study per our interrupted time series analyses are comparable to the one randomized controlled trial that evaluated a CDS intervention to improve VTE prophylaxis [10]. In addition, our revision to the CDS intervention where we linked the risk assessment to the prophylaxis order set grid had the intended effect of improving the concordance between the users’ risk assessments (i.e. what they said they would order) and the prophylaxis actually ordered.

Advantages to the approach we employed are several. First, we were able to avoid the use of pop-up alerts and the risk for subsequent alert fatigue [18]. Second, we were able to create our order set within a commercial EHR used by more than 300 academic and community healthcare institutions in and outside the U.S.; thus, our intervention can be utilized by many other institutions either using the same or a similar EHR. Third, we were able to reach all hospitalized populations across multiple hospitals in an academic health care system, instead of select populations [8,11,12] or a single hospital [17] as investigated in many other studies.

Despite our increase in “recommended” and “any” VTE prophylaxis, the VTE event rates across our overall study population increased (2.0% to 2.1% to 2.2%; p = 0.02). There are a number of potential explanations. First, improvements in coding may have increased the number of VTE events captured. To understand the impact of coding, we performed a subanalysis where we excluded patients with VTE codes listed as POA; this resulted in no significant difference in VTE rates across our study periods (1.3% vs. 1.3% vs. 1.3%; p = 0.80), suggesting that increased coding of VTE diagnoses that were actually POA may have resulted in a seeming increase in overall VTE rates across our study periods. Second, there may have been an increase in the VTE risk of our study population over time. Although Table 1 indicated few clinically significant changes, a post hoc analysis of the proportion of our patients receiving peripherally inserted central catheters or midlines during their hospital stay suggested an increase in the use of such lines (6.9% vs. 7.1% vs. 8.2% for study periods 1, 2 and 3 respectively; p < 0.01). This could increase the risk of line-associated (LA) VTE, and is an important VTE risk factor as routine prophylaxis has not been consistently shown to be effective in reducing the rate of LA VTE [23-27]. Unfortunately, codes differentiating LA VTE from other VTE were not used in our health system until the end of our study period. Third, our measure of VTE prophylaxis was defined by the order selected at time of admission for mechanical prophylaxis, and an order and at least one administered dose for pharmacologic prophylaxis. Thus, if mechanical prophylaxis was not routinely applied to patients, or if subsequent doses of pharmacologic prophylaxis were routinely missed or not administered, then we wouldn’t expect to see an impact of the intervention on VTE rates. Finally, our hospital system switched LMWH products from enoxaparin to dalteparin in December 2009. It is unclear what impact this switch may have had on VTE rates, although limited evidence suggests no differences between the two products [28].

Despite the lack of improvement in VTE rates over time in our overall study population, the analysis of a prespecified surgical subset of our sample as defined by the AHRQ PSI for public reporting purposes [20] demonstrated a statistically significant reduction in DVT and overall VTE events. Increased awareness of and emphasis on VTE prophylaxis in this subpopulation as a result of the AHRQ PSI metric may have caused the greater improvements in VTE prophylaxis demonstrated in this subpopulation. Specifically, those services described as the “other surgical services” in our study had the greatest increases overall in both recommended prophylaxis (with an approximate absolute increase of 30% over the study period) and pharmacologic prophylaxis (with an approximate absolute increase of 20% over the study period). Such large increases in prophylaxis may have resulted in the reduction in VTE events demonstrated in this subpopulation. The findings from this subpopulation also suggest that our intervention may have more impact in populations at higher risk of VTE (such as the surgical population defined by the AHRQ measure), the effect of which is blunted when the entire hospital population is analyzed.

The increase in PE demonstrated in the subanalysis of the AHRQ PSI surgical population is more challenging to explain. To our knowledge, there were no changes in our patient population over time that would have increased their risk of PE without increasing their risk of DVT. Moreover, there were no changes in documentation codes for PE during the study period. It is possible that increased national awareness of VTE may have increased providers’ clinical suspicion for PE out of proportion to their clinical suspicion for DVT, and this may have resulted in increased testing for and diagnosis of PE. In addition, recent publications have suggested that the improved sensitivities of new CT technology and protocols have resulted in the diagnosis of PEs previously undetected and unsuspected [29-31]. Such improvements may have contributed to the increased incidence of PE demonstrated in the US in the last two decades [29,30], as well as the increased incidence of PE demonstrated across the three years of this study.

When accounting for VTE “present on admission”, our overall health system VTE rate of 1.3% is reasonably consistent with those we would expect in an inpatient population treated with VTE prophylaxis. Studies suggest that rates can be as low as 0.8% for symptomatic DVT and 0.8% for PE in nonsurgical patients [2], and as low as 1.3% for symptomatic DVT and 0.6% for PE (for a total VTE rate of 1.8%) in patients undergoing major orthopedic surgery [3].

Our study has limitations. First, our simple pre-post design does not fully account for secular changes that may result in improvements in VTE prophylaxis and reductions in VTE events, such as greater awareness of the importance of VTE prophylaxis among providers. However, our use of multiple time points before and after the intervention, including modeling to adjust for secular trends in the data, allow us to estimate with more certainty the change in VTE prophylaxis directly attributable to the intervention. Second, our measure of VTE events and bleeding are based on administrative data and are dependent on appropriate documentation and coding. However, a prior study suggests that administrative data can have reasonable sensitivity for VTE, with sensitivities for DVT and PE of 87% and 78% respectively [32]. Another study reported reasonable test characteristics for the bleeding codes we used, with 93% sensitivity and 88% specificity for “any” bleeding, and 94% sensitivity and 83% specificity for “major” bleeding [21]. However, this suggests that some bleeds we attributed to VTE prophylaxis were actually due to other causes, and that some bleeds due to VTE prophylaxis were missed by our analysis. In addition, our definition of hospital-acquired VTE was limited by the lack of documentation in our records regarding VTE “present on admission” for the first six months of the baseline period of our study for Hospital A and Hospital C, and for the entire baseline period of our study for Hospital B. Thus, our primary analyses could not discriminate as well between VTE “present on admission” and those acquired during the hospital stay. This along with increased coding for VTE in general may have accounted for the seeming increases in our VTE events over time as described above, increases which were NOT demonstrated in our subanalysis using only admissions with appropriate POA documentation. Third, our measure of why providers declined VTE prophylaxis is limited by provider self report. Fourth, our study did not examine the effect of our CDS intervention on VTE events after hospital discharge, where many such events may occur. Lastly, our CDS intervention was based on a national guideline [1] that has since been updated [3,33,34].

## Conclusions

Our analysis demonstrated significant increases in VTE prophylaxis that were associated with a CDS intervention integrated into an electronic admission order across a multi-hospital academic health system. The intervention was also associated with increased VTE rates in the overall study population, but a subanalysis using only admissions with appropriate POA documentation suggested no change in VTE rates, and a prespecified analysis of a surgical subset of our sample as defined by the AHRQ PSI for public reporting purposes suggested reduced VTE. This CDS was created in a commonly used commercial EHR and is thus scalable across other institutions with similar systems.

## Competing interests

This study received support from an Institutional Clinical and Translational Science Award from the NIH (5-UL1RR024134-02). The authors declare that they have no competing interests.

## Authors’ contributions

CAU participated in the conception, coordination, and design of the study as well as data analysis and interpretation, and drafted and revised the manuscript. AH participated in the study design, data analysis and interpretation, and acquired the data. JC participated in the study design and data analysis and interpretation, and drafted part of the manuscript. MW and TEHH participated in study design, data analysis and interpretation, and contributed critical revisions to the draft manuscript. All authors read and approved the final manuscript.

## Pre-publication history

The pre-publication history for this paper can be accessed here:

http://www.biomedcentral.com/1472-6947/12/92/prepub
